# AI performance by mammographic density in a retrospective cohort study of 99,489 participants in BreastScreen Norway

**DOI:** 10.1007/s00330-024-10681-z

**Published:** 2024-03-25

**Authors:** Marie Burns Bergan, Marthe Larsen, Nataliia Moshina, Hauke Bartsch, Henrik Wethe Koch, Hildegunn Siv Aase, Zhanbolat Satybaldinov, Ingfrid Helene Salvesen Haldorsen, Christoph I. Lee, Solveig Hofvind

**Affiliations:** 1 Section for Breast Cancer Screening, Cancer Registry of Norway, Norwegian Institute of Public Health, P.O. Box 5313, 0304 Oslo, Norway; 2 Department of Radiology, Mohn Medical Imaging and Visualization Centre (MMIV), Haukeland University Hospital, Bergen, Norway; 3 Department of Radiology, Stavanger University Hospital, Stavanger, Norway; 4 Faculty of Health Sciences, University of Stavanger, Stavanger, Norway; 5 Department of Radiology, Haukeland University Hospital, Bergen, Norway; 6 Section for Radiology, Department of Clinical Medicine, University of Bergen, Bergen, Norway; 7 Department of Radiology, University of Washington School of Medicine, Seattle, WA USA; 8 Department of Health Systems and Population Health, University of Washington School of Public Health, Seattle, WA USA; 9 Department of Health and Care Sciences, Faculty of Health Sciences, UiT The Arctic University of Norway, Tromsø, Norway

**Keywords:** Breast cancer, Mammography, Screening, Artificial intelligence, Breast density

## Abstract

**Objective:**

To explore the ability of artificial intelligence (AI) to classify breast cancer by mammographic density in an organized screening program.

**Materials and method:**

We included information about 99,489 examinations from 74,941 women who participated in BreastScreen Norway, 2013–2019. All examinations were analyzed with an AI system that assigned a malignancy risk score (AI score) from 1 (lowest) to 10 (highest) for each examination. Mammographic density was classified into Volpara density grade (VDG), VDG1–4; VDG1 indicated fatty and VDG4 extremely dense breasts. Screen-detected and interval cancers with an AI score of 1–10 were stratified by VDG.

**Results:**

We found 10,406 (10.5% of the total) examinations to have an AI risk score of 10, of which 6.7% (704/10,406) was breast cancer. The cancers represented 89.7% (617/688) of the screen-detected and 44.6% (87/195) of the interval cancers. 20.3% (20,178/99,489) of the examinations were classified as VDG1 and 6.1% (6047/99,489) as VDG4. For screen-detected cancers, 84.0% (68/81, 95% CI, 74.1–91.2) had an AI score of 10 for VDG1, 88.9% (328/369, 95% CI, 85.2–91.9) for VDG2, 92.5% (185/200, 95% CI, 87.9–95.7) for VDG3, and 94.7% (36/38, 95% CI, 82.3–99.4) for VDG4. For interval cancers, the percentages with an AI score of 10 were 33.3% (3/9, 95% CI, 7.5–70.1) for VDG1 and 48.0% (12/25, 95% CI, 27.8–68.7) for VDG4.

**Conclusion:**

The tested AI system performed well according to cancer detection across all density categories, especially for extremely dense breasts. The highest proportion of screen-detected cancers with an AI score of 10 was observed for women classified as VDG4.

**Clinical relevance statement:**

Our study demonstrates that AI can correctly classify the majority of screen-detected and about half of the interval breast cancers, regardless of breast density.

**Key Points:**

*• Mammographic density is important to consider in the evaluation of artificial intelligence in mammographic screening.*

*• Given a threshold representing about 10% of those with the highest malignancy risk score by an AI system, we found an increasing percentage of cancers with increasing mammographic density.*

*• Artificial intelligence risk score and mammographic density combined may help triage examinations to reduce workload for radiologists.*

**Supplementary Information:**

The online version contains supplementary material available at 10.1007/s00330-024-10681-z.

## Introduction

Most European countries have implemented mammographic screening programs over the last decades, aiming to reduce breast cancer mortality [1, 2]. In Norway, the national screening program for breast cancer, BreastScreen Norway, started in 1996 and invites all women aged 50–69 to two-view mammographic screening biennially [3]. All examinations are independently double read, and more than 99% of the women are not diagnosed with breast cancer at screening.

The target group for mammographic screening is asymptomatic women with average risk of breast cancer [4]. However, breast cancer risk varies substantially across the general screening population [5]. Mammographic breast density, i.e., the proportion of fibroglandular tissue in the breast relative to the proportion of fatty tissue, is an independent risk factor for breast cancer, and women with extremely dense breasts reportedly have 4–6 times higher risk of breast cancer compared to women with fatty breasts [6, 7]. Further, the sensitivity of mammographic screening for these women is below 70% compared to 85–90% for women with fatty breasts, due to a masking effect [8].

In March 2022, screening recommendations for women with extremely dense breasts were published by the European Society of Breast Imaging (EUSOBI) [9] and further supported by several EU groups [10, 11]. These risk-based screening recommendations stated that women with extremely dense breasts should be offered supplemental screening with breast MRI every 2 to 4 years [9]. Other suggestions to detect cancers in dense breasts are additional ultrasound [12] and contrast-enhanced spectral mammography [13] as well as annual mammography [14, 15].

The introduction of artificial intelligence (AI) as an adjunct tool for cancer detection in mammographic screening has shown promising results in retrospective and prospective studies [16–26]. However, results from studies reporting the accuracy of AI breast cancer risk scores based on mammographic density categories are sparse. In a retrospective study from Norway with cancer-enriched data from a screening setting, AI scored the highest risk of malignancy on all cancer cases in women with extremely dense breasts [13]. Knowledge about the AI risk scores and cancer detection across all mammographic densities is thus important, especially for women with extremely dense breasts who already experience lower screening sensitivity.

In this study, we aimed to analyze the ability of an AI system to classify breast cancers by mammographic density in BreastScreen Norway, an organized screening environment that utilizes independent double reading. Further, we analyzed histopathological characteristics of screen-detected and interval cancers by AI score.

## Materials and methods

**Fig. 1:**
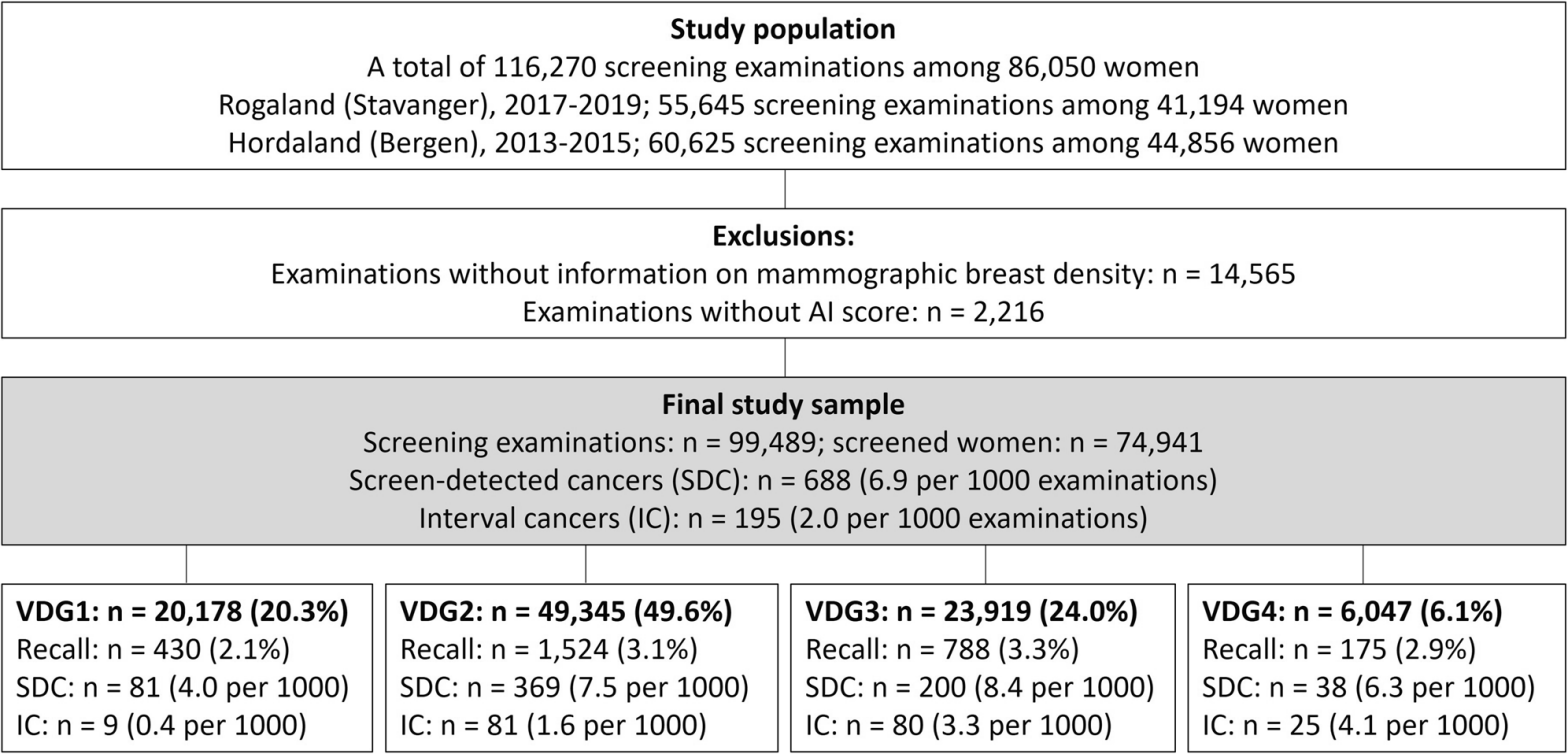
Flow chart of the study sample and final study sample stratified by Volpara density grade (VDG) 1–4, including recall, screen-detected cancers (SDC), and interval cancers (IC)

This retrospective cohort study included images from digital mammography and screening data obtained from two breast centers, Rogaland (Stavanger) and Hordaland (Bergen), during the period from January 2013 to December 2019 (Fig. 1). The study was approved by the Regional Committee for Medical and Health Research Ethics (REC # 2018/2574) and has a legal basis in accordance with Articles 6 (1) (e) and 9 (2) (j) of the General Data Protection Regulation (GDPR). The data was disclosed with legal basis in the Cancer Registry Regulations section 3–1 and the Personal Health Data Filing System Act section 19 a to 19 h [27, 28]. Parts of the study sample have been used in previous publications, but for different study objectives [13, 15].

In BreastScreen Norway, all women aged 50–69 years are invited to two-view biennial digital mammographic screening. All mammograms are interpreted independently by two breast radiologists. Each breast is given an interpretation score between 1 and 5, where 1 indicates negative for abnormality; 2, probably benign; 3, intermediate suspicion; 4, probably malignant; and 5, high suspicion of malignancy. If either radiologist assigns a score 2 or higher, the examination is discussed in a consensus meeting to determine whether or not to recall the woman for further assessment [3].

Examinations performed in Hordaland 2016–2019 were excluded due to a tomosynthesis trial in Bergen [29]. All women were screened with GE Senographe Pristina or GE Senographe Essential. After excluding examinations without information on mammographic breast density and examinations without AI score, the final study sample included 99,489 screening examinations (Fig. 1).

### AI risk score and mammographic breast density

The commercial AI system Transpara version 1.7.0, developed by ScreenPoint Medical, was used to score mammography exams in this study [18, 30]. The AI system uses convolutional neural networks to analyze mammographic images and provide one malignancy risk score for each view of each breast [18]. The algorithm has been trained on mammograms from different screening programs and vendors. In this study, the highest score of all views was referred to as the overall AI score of an examination. The AI score ranged from 1 to 10; a score of 1–7 was considered low risk of breast cancer, a score of 8–9 intermediate risk, and a score of 10 was considered high risk of breast cancer, as prescribed by ScreenPoint Medical.

Volumetric breast density was obtained from the automated software Volpara, versions 1.5.0 and 1.5.4.0 [31]. Examinations were classified into four Volpara density grades (VDG) based on the maximum percentage of volumetric breast density between the left and right breast per examination. Examinations with a volumetric density of ≤ 3.4% were classified as VDG1, 3.5–7.4% as VDG2, 7.5–15.4% as VDG3, and ≥ 15.5% as VDG4 [32]. The classification system is analogous to the four-category BI-RADS, 5th edition system, categories: almost entirely fatty, scattered fibroglandular tissue, heterogeneously dense tissue, and extremely dense tissue.

### Breast cancer and histopathological tumor characteristics

A negative screening examination was defined as an examination scored 1 by both radiologists, an examination with score ≥ 2, but dismissed at consensus, or a recall from screening with negative diagnostic outcome (false-positive screening result). Screen-detected cancer was defined as histologically verified ductal carcinoma in situ (DCIS) or invasive breast cancer diagnosed after a recall assessment and within 6 months after the screening examination. Interval cancer was defined as DCIS or invasive breast cancer diagnosed within 24 months of a negative screening result or 6–24 months after a false-positive screening result [3]. For the interval cancer cases, mammograms from the screening examination prior to diagnosis were scored by the AI system.

Histopathological tumor characteristics were collected for invasive screen-detected and interval cancers, and included histologic type (invasive carcinoma of no special type, invasive lobular carcinoma, invasive tubular carcinoma, and other invasive carcinomas), tumor diameter in millimeters, histologic grade 1–3 [33], lymph node involvement, and immunohistochemical subtypes. Subtypes were categorized into Luminal A-like, Luminal B-like Her2 + , Luminal B-like Her2 − , Her2 + , and triple negative [34].

### Statistical analysis

Using descriptive analyses, we assessed the overall performance of the AI system in successfully categorizing cancers into high-risk categories, stratified by VDG. We provided frequencies and percentages of all screening examinations, examinations with a negative screening result, screen-detected cancers, interval cancers, and all cancers combined, stratified by AI scores 1–10. Cumulative frequencies and percentages were presented for screen-detected and interval cancers, and the percentage of screen-detected + interval cancers among all cancers was presented by cumulative AI score. 95% confidence intervals were computed using the exact binomial distribution. The percentages of screen-detected or screen-detected + interval cancer could be considered the sensitivity of the AI system at a given threshold. Histopathological tumor characteristics of invasive screen-detected and interval cancers (median tumor diameter with interquartile range (IQR) and numbers and percentages for lymph node status, grade, and immunohistochemical subtypes) are presented for AI score 10 and AI score 1–9. Associations between each tumor characteristic and AI score were tested with bivariate tests. Frequencies and percentages of all examinations, and for screen-detected cancers separately, were presented by VDG and by AI score 1 to 10. For screen-detected and interval cancers with an AI score of 10, the percentages of cases classified into each density category were graphically presented. Further, theoretical triage scenarios for the AI system were explored based on combinations of AI scores and mammographic density. All statistical analyses were performed in Stata version 17.0.

## Results

### Overall performance

The final study sample included data from 99,489 screening examinations among 74,941 women, including 883 breast cancers (688 screen-detected, 195 interval cancers) (Fig. 1). Among all screening examinations, 20.5% (20,378/99,489, 95% CI, 20.2–20.7) had an AI score of 1, and 10.5% (10,406/99,489, 95% CI,10.3–10.7) had an AI score of 10 (Table 1). We found that 89.7% (617/688, 95% CI, 87.2–91.9) of the screen-detected and 44.6% (87/195, 95% CI, 37.5–51.9) of the interval cancers were assigned an AI score of 10 (Table 1) and 5.9% (617/10,406, 95% CI, 5.5–6.4) of the examinations assigned an AI score of 10 to be screen-detected cancer (Table 2). In total, the sensitivity of the AI system was 79.7% (704/883, 95% CI, 76.9–82.3). In Stavanger, 94.8% (290/306) of the screen-detected and 42.9% (42/98) of the interval cancers had an AI score of 10, while in Bergen, the corresponding percentages were 85.6% (327/382) and 46.4% (45/97), respectively (Supplementary Table 1).

**Table 1:** Frequencies (*n*) and percentages (%) with 95% confidence intervals (CI) of all screening examinations, examinations with a negative screening result, screen-detected cancers, interval cancers, and all cancers combined, stratified by AI score 1–10

AI score	All screening examinations			Examinations with negative screening results			Screen-detected cancers			Interval cancers			Screen-detected and interval cancers		
	*n*	%	(95% CI)	*n*	%	(95% CI)	*n*	%	(95% CI)	*n*	%	(95% CI)	*n*	%	(95% CI)
1	20,378	20.5	(20.2–20.7)	20,362	20.7	(20.6–20.9)	3	0.4	(< 0.1–1.2)	13	6.7	(3.6–11.1)	16	1.8	(1.0–2.9)
2	7637	7.7	(7.5–7.8)	7628	7.7	(7.6–7.9)	2	0.3	(< 0.1–1.0)	7	3.6	(1.5–7.3)	9	1.0	(0.5–1.9)
3	9562	9.6	(9.4–9.8)	9553	9.7	(9.5–9.9)	3	0.4	(< 0.1–1.2)	6	3.1	(1.1–6.6)	9	1.0	(0.5–1.9)
4	8961	9.0	(8.8–9.2)	8946	9.1	(8.9–9.3)	4	0.6	(0.2–1.5)	11	5.6	(2.8–9.9)	15	1.7	(1.0–2.5)
5	8612	8.7	(8.5–8.8)	8602	8.7	(8.5–8.9)	3	0.4	(< 0.1–1.2)	7	3.6	(1.5–7.3)	10	1.1	(0.5–2.1)
6	7661	7.7	(7.5–7.9)	7646	7.8	(7.6–7.9)	2	0.3	(< 0.1–1.0)	13	6.7	(3.6–11.1)	15	1.7	(1.0–2.5)
7	7636	7.7	(7.5–7.8)	7625	7.7	(7.6–7.9)	4	0.6	(0.2–1.5)	7	3.6	(1.5–7.3)	11	1.3	(0.6–2.2)
8	8510	8.6	(8.4–8.7)	8469	8.6	(8.4–8.8)	21	3.1	(1.9–4.6)	20	10.3	(6.4–15.4)	41	4.6	(3.4–6.2)
9	10,126	10.2	(10.0–10.4)	10,073	10.2	(10.0–10.4)	29	4.2	(2.8–6.0)	24	12.3	(8.0–17.8)	53	6.0	(4.5–7.8)
10	10,406	10.5	(10.3–10.7)	9702	9.8	(9.7–10.0)	617	89.7	(87.2–91.9)	87	44.6	(37.5–51.9)	704	79.7	(76.9–82.3)
Total	99,489	100.0	(-)	98,606	100.0	(-)	688	100.0	(-)	195	100.0	(-)	883	100.0	(-)

**Table 2:** Cumulative frequencies (*n*) of screening examinations, cumulative *n* and percentages (%) with 95% confidence intervals (CI) of screen-detected cancers and interval cancers, and the percentage of screen-detected + interval cancer among all cancers detected (sensitivity), by AI score 1–10

AI score	All screening examinations	Screen-detected cancers*			Interval cancers^§^			Sensitivity of screen-detected and interval cancers^#^		
	*n*	*n*	%	(95% CI)	*n*	%	(95% CI)	*n*	%	(95% CI)
≥ 10	10,406	617	5.9	(5.5–6.4)	87	0.8	(0.7–10.3)	704	79.7	(76.9–82.3)
≥ 9	20,532	646	3.1	(2.9–3.4)	111	0.5	(0.4–0.7)	757	85.7	(83.2–88.0)
≥ 8	29,042	667	2.3	(2.1–2.5)	131	0.5	(0.4–0.5)	798	90.4	(88.2–92.2)
≥ 7	36,678	671	1.8	(1.7–2.0)	138	0.4	(0.3–0.4)	809	91.6	(89.6–93.3)
≥ 6	44,339	673	1.5	(1.4–1.6)	151	0.3	(0.3–0.4)	824	93.3	(91.5–94.9)
≥ 5	52,951	676	1.3	(1.1–1.4)	158	0.3	(0.3–0.3)	834	94.5	(92.7–95.9)
≥ 4	61,912	680	1.1	(1.0–1.2)	169	0.3	(0.2–0.3)	849	96.1	(94.7–97.3)
≥ 3	71,474	683	1.0	(0.9–1.0)	175	0.2	(0.2–0.3)	858	97.2	(95.8–98.2)
≥ 2	79,111	685	0.9	(0.9–0.9)	182	0.2	(0.2–0.3)	867	98.2	(97.1–99.0)
≥ 1	99,489	688	0.7	(0.6–0.7)	195	0.2	(0.2–0.2)	883	100.0	(-)

^*^Screen-detected cancers divided by all screening examinations by cumulative AI score

^§^Interval cancers divided by all screening examinations by cumulative AI score

^#^Number of screen-detected and interval cancers in the different AI score groups divided by the total number of cancers

### Histopathological tumor characteristics

For an AI score of 10, 18.6% (115/617) of the screen-detected cancers was DCIS while it was 5.6% (4/71, *p* = 0.01) for those with an AI score of 1–9 (Table 3). For interval cancers, the percentages were 6.9% (6/87) for an AI score of 10 and 7.4% (8/108, *p* = 0.89) for an AI score of 1–9. Tumor diameter and Van Nuys grade for screen-detected and interval DCIS included a small number of cases and are shown in Supplementary Table 2.

**Table 3:** Histopathological tumor characteristics of invasive screen-detected and interval cancers with AI score 10 and AI score 1–9

	Screen-detected cancers					Interval cancers				
	AI score 10, *n* = 617		AI score 1–9, *n* = 71		*p*-value*	AI score 10, *n* = 87		AI score 1–9, *n* = 108		*p*-value*
	*n*	%	*n*	%		*n*	%	*n*	%	
Ductal carcinoma in situ	115	18.6	4	5.6	0.01	6	6.9	8	7.4	0.89
Invasive cancers	502	81.4	67	94.4		81	93.1	100	92.6	
Invasive cancers										
Histologic type					0.04					0.45
Invasive carcinoma of no special type	429	85.5	57	85.1		60	74.1	76	76.0	
Invasive lobular carcinoma	46	9.2	5	7.5		17	21.0	23	23.0	
Invasive tubular carcinoma	-	-	-	-		-	-	-	-	
Other invasive carcinomas	27	5.4	5	7.5		4	4.9	1	1.0	
Tumor diameter (mm), median (IQR)	14 (10–21)		13 (8–16)		0.01	19 (13–30)		24 (15–30)		0.80
Information not available	13		1			7		15		
Histologic grade					0.83					0.17
Grade 1	166	34.2	24	36.9		14	18.9	16	18.8	
Grade 2	208	42.8	28	43.1		36	48.6	30	35.3	
Grade 3	112	23.0	13	20.0		24	32.4	39	45.9	
Information not available	16		2			7		15		
Lymph node positive	81	16.5	8	12.3	0.39	27	35.5	28	30.8	0.52
Information not available	11		2			5		9		
Immunohistochemical subtypes					0.48					0.11
Luminal A-like	251	52.8	37	60.7		26	34.2	31	34.1	
Luminal B-like, Her2 −	113	23.8	15	24.6		16	21.1	25	27.5	
Luminal B-like, Her2 +	60	12.6	6	9.8		20	26.3	10	11.0	
Her2 +	16	3.4	-	-		4	5.3	7	7.7	
Triple negative	35	7.4	3	4.9		10	13.2	18	19.8	
Information not available	27		6			5		9		

^*^Overall association between each tumor characteristic variable and AI score (10 vs. 1–9) was tested with a bivariate test for continuous or categorical outcome as appropriate

Among screen-detected cancers with an AI score of 10, 81.4% (502/617) were invasive (Table 3). For these cancers, median tumor diameter was 14 mm (IQR, 10–21), 23.0% (112/486) were histologic grade 3, 16.5% (81/491) were lymph node positive, and 7.4% (35/475) triple negative. For screen-detected cancers with an AI score of 1–9, 94.4% (67/71, *p* = 0.01) were invasive, median tumor diameter was 13 mm (IQR, 8–16, *p* = 0.01), 20.0% (13/65, *p* = 0.83) histologic grade 3, 12.3% (8/65, *p* = 0.39) lymph node positive, and 4.9% (3/61, *p* = 0.48) triple negative.

For interval cancers, 93.1% (81/87) with an AI score of 10 were invasive versus 92.6% (100/108, *p* = 0.89) for AI score 1–9 (Table 3). Among the cases with an AI score of 10, median tumor diameter was 19 mm (IQR, 13–30) and 32.4% (24/74) were histologic grade 3 compared to median tumor diameter of 24 mm (IQR, 15–30, *p* = 0.80) and 45.9% (39/85, *p* = 0.17) histologic grade 3 for those with an AI score of 1–9. A total of 35.5% (27/76) of interval cancers with an AI score of 10 were lymph node positive and 13.2% (10/76) were triple negative. The percentages for lymph node positive and triple negative were 30.8% (28/91, *p* = 0.52) and 19.8% (18/91, *p* = 0.11), respectively, for interval cancer cases with an AI score of 1–9.

### Mammographic density

In our study sample, 20.3% (20,178/99,489) of the examinations were classified as VDG1, 49.6% (49,345/99,489) as VDG2, 24.0% (23,919/99,489) as VDG3, and 6.1% (6047/99,489) as VDG4 (Fig. 1). The highest rate of screen-detected cancers was observed for VDG3 (8.4 per 1000), while the lowest observed was for VDG1 (4.0 per 1000). The highest interval cancer rate (4.1 per 1000) was observed for VDG4 while the lowest rate was observed for VDG1 (0.4 per 1000).

Among the examinations classified as VDG1, 7.4% (1484/20,178) had the highest AI score of 10 (Table 4). The percentage was 11.0% (5405/49,345) for those with VDG2, 11.3% (2705/23,919) for VDG3, and 13.4% (812/6047) for VDG4. In Stavanger and Bergen, 12.7% (417/3276) and 14.3% (395/2771) of those with VDG4 had an AI score of 10, respectively (Supplementary Table 3).

**Table 4:** Frequencies (*n*) and percentages (%) with 95% confidence intervals (CI) of screening examinations with AI score 1–10, stratified by volumetric breast density (VDG1, VDG2, VDG3, and VDG4)

AI score	VDG1			VDG2			VDG3			VDG4		
	*n*	%	(95% CI)	*n*	%	(95% CI)	*n*	%	(95% CI)	*n*	%	(95% CI)
1	6522	32.3	(31.7–33.0)	9235	18.7	(18.4–19.1)	3532	14.8	(14.3–15.2)	1089	18.0	(17.0–19.0)
2	1270	6.3	(6.0–6.6)	3639	7.4	( 7.1–7.6)	2142	9.0	(8.6–9.3)	586	9.7	(9.0–10.5)
3	2122	10.5	(10.1–10.9)	4784	9.7	(9.4–10.0)	2146	9.0	(8.6–9.3)	510	8.4	(7.7–9.2)
4	1749	8.7	(8.3–9.1)	4570	9.3	(9.0–9.5)	2188	9.1	(8.8–9.5)	454	7.5	(6.9–8.2)
5	1563	7.7	(7.4–8.1)	4300	8.7	(8.5–9.0)	2287	9.6	(9.2–9.9)	462	7.6	(7.0–8.3)
6	1307	6.5	(6.1–6.8)	3904	7.9	(7.7–8.2)	2030	8.5	(8.1–8.8)	420	6.9	(6.3–7.6)
7	1309	6.5	(6.2–6.8)	4896	7.9	(7.7–8.1)	1985	8.3	(8.0–8.7)	446	7.4	(6.7–8.0)
8	1305	6.5	(6.1–6.8)	4474	9.1	(8.8–9.3)	2194	9.2	(8.8–9.5)	537	8.9	(8.2–9.6)
9	1547	7.7	(7.3–8.0)	5138	10.4	(10.1–10.7)	2710	11.3	(10.9–11.7)	731	12.1	(11.3–12.9)
10	1484	7.4	(7.0–7.7)	5405	11.0	(10.7–11.2)	2705	11.3	(10.9–11.7)	812	13.4	(12.6–14.3)
Total	20,178	100.0	(-)	49,345	100.0	(-)	23,919	100.0	(-)	6047	100.0	(-)

**Fig. 2:**
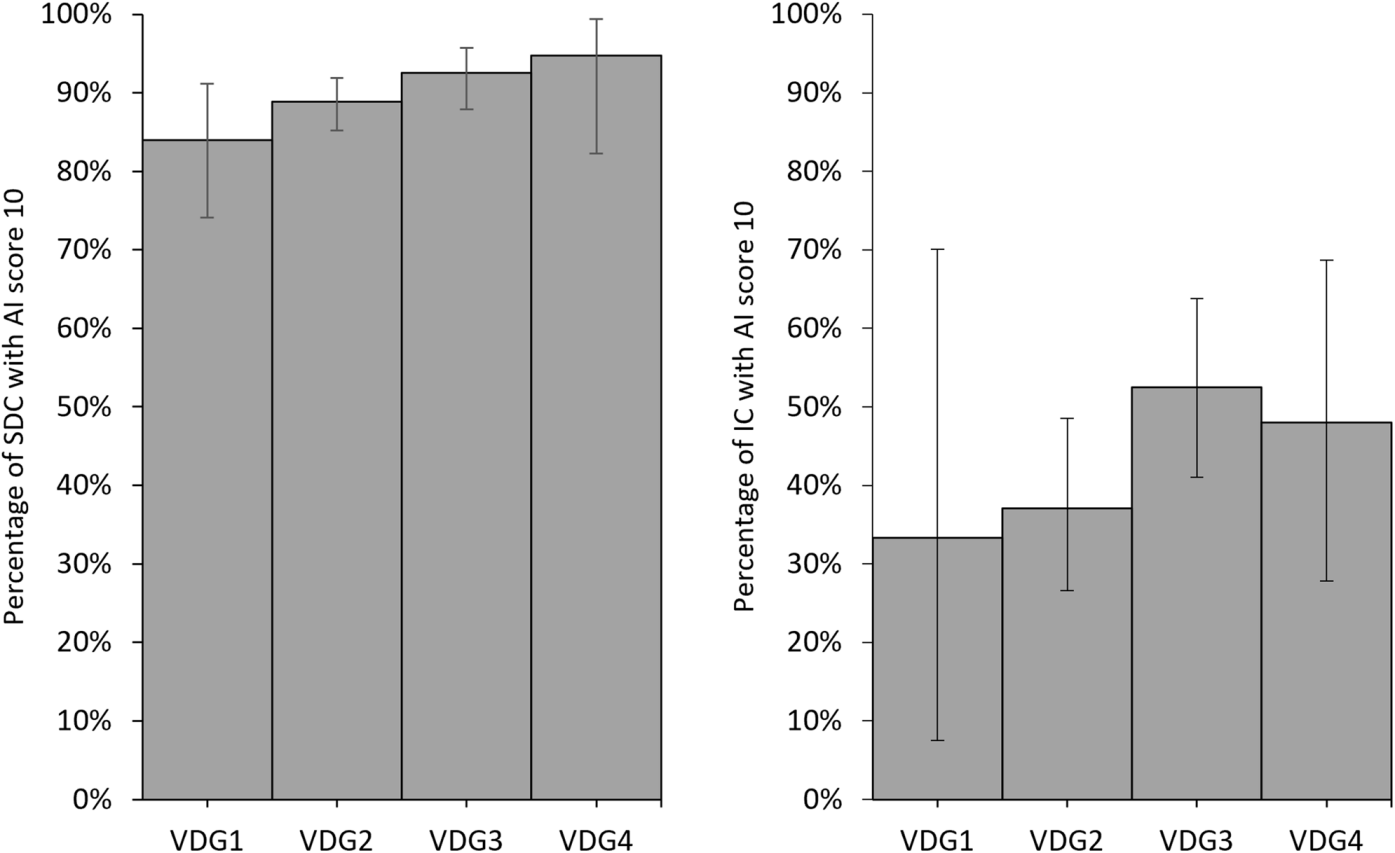
Percentage with 95% confidence intervals (CI) of screen-detected (SDC) and interval cancers (IC) with AI score 10, stratified by VDG1, VDG2, VDG3, and VDG4

For screen-detected cancers, 84.0% (68/81, 95% CI, 74.1–91.2) of the women classified as VDG1 had an AI score of 10 (Table 5, Fig. 2). The percentage was 88.9% (328/368, 95% CI, 85.2–91.9) for women with VDG2, 92.5% (185/200, 95% CI, 87.9–95.7) for VDG3, and 94.7% (36/38, 95% CI, 82.3–99.4) for VDG4. Of the interval cancer cases, 33.3% (3/9, 95% CI, 7.5–70.1) of women with VDG1, 37.0% (30/81, 95% CI, 26.6–48.5) with VDG2, 52.5% (42/80, 95% CI, 41.0–63.8) with VDG3, and 48.0% (12/25, 95% CI, 27.8–68.7) with VDG4 had the highest AI score of 10 (Supplementary Table 4, Fig. 2).

**Table 5:** Frequencies (*n*) and percentages (%) of screen-detected cancers with AI score 1–10, stratified by volumetric breast density (VDG1, VDG2, VDG3, and VDG4)*

AI Score	VDG1		VDG2		VDG3		VDG4	
	*n*	%	*n*	%	*n*	%	*n*	%
1	1	1.2	2	0.5	0	-	0	-
2	0	-	1	0.3	1	0.5	0	-
3	0	-	1	0.3	2	1.0	0	-
4	0	-	4	1.1	0	-	0	-
5	2	2.5	1	0.3	0	-	0	-
6	1	1.2	0	-	1	0.5	0	-
7	3	3.7	0	-	1	0.5	0	-
8	2	2.5	16	4.3	3	1.5	0	-
9	4	4.9	16	4.3	7	3.5	2	5.3
10	68	84.0	328	88.9	185	92.5	36	94.7
Total	81	100.0	369	100.0	200	100.0	38	100.0

^*^Due to the small numbers, 95% confidence intervals or *p*-values were not estimated

### Triaging examinations based on AI score and mammographic density

In a hypothetical triage setting, where examinations with an AI score of 1–5 are classified as negative and excluded from the radiologist interpretive workflow, the reader volume would be reduced by 55.4% (55,150/99,489), while 2.2% (15/688) of the screen-detected cancers would be classified as negative (Table 1). Defining AI scores 1–7 as negative, the reading volume would be reduced by 70.8% (70,447/99,489), at the cost of classifying 3.1% (21/688) of the screen-detected cancers as negative (Table 1).

**Fig. 3:**
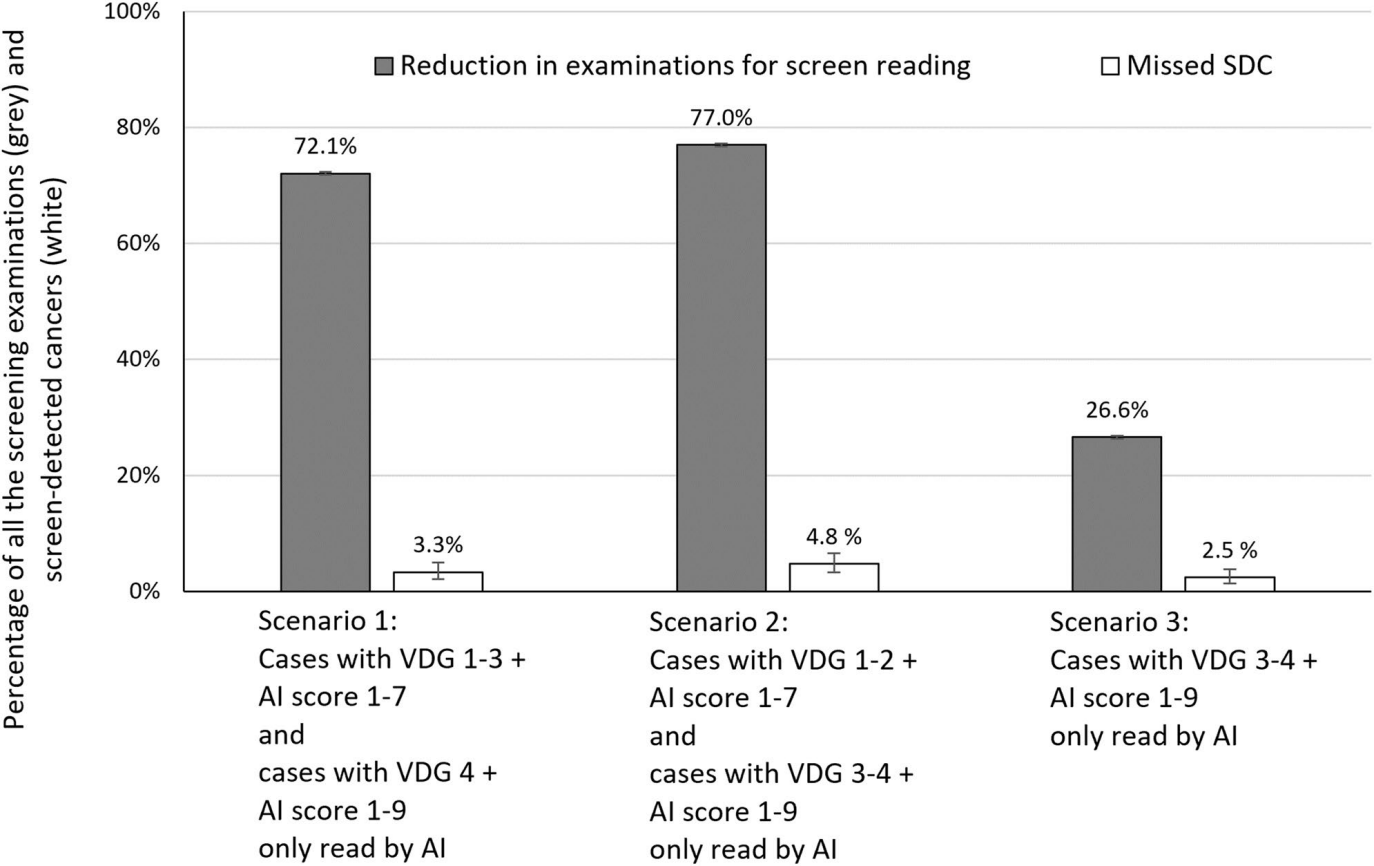
Percentage with 95% confidence intervals (CI) of reduction in examinations for screen reading (gray) and of missed screen-detected cancers (white) for three different triaging scenarios

Hypothetically, if examinations were triaged based on AI scores and mammographic density, where VDG1–3 examinations with an AI score of 1–7 (low risk) and VDG4 examinations with an AI score of 1–9 (low and intermediate risk) were excluded from the radiologists’ workflow, then the reading volume would be reduced by 72.1% (71,715/99,489, 95% CI, 71.8–72.4) at the cost of 3.3% (23/688, 95% CI, 2.1–5.0) of the screen-detected cancers classified as negative (scenario 1 in Fig. 3, Table 4, Table 5). The reader volume would be reduced by 77.0% (76,619/99,489, 95% CI, 76.7–77.3) at the cost of classifying 4.8% (33/688, 95% CI, 3.3–6.7) of the screen-detected cancers as negative if VDG1–2 examinations with an AI score of 1–7 and VDG3–4 examinations with an AI score of 1–9 were interpreted only by AI (scenario 2 in Fig. 3). In a setting where VDG3–4 examinations with an AI score of 1–9 were interpreted only by AI, 26.6% (26,449/99,489, 95% CI, 26.3–26.9) of the examinations would be removed from the radiologist’s workflow and 2.5% (17/688, 95% CI, 1.4–3.9) of the screen-detected cancers would be classified as negative (scenario 3 in Fig. 3).

## Discussion

In this retrospective cohort study including nearly 100,000 examinations performed with mammography equipment from GE Healthcare, we found that 89.7% (617/688) of screen-detected cancers and 44.6% (87/195) of the interval cancers were assigned an AI score of 10, implicating the highest suspicion of malignancy. For women with extremely dense breasts (VDG4), 94.7% (36/38) of the screen-detected cancers and 48.0% (12/25) of the interval cancers were given an AI score of 10. For VDG2, the corresponding numbers were 88.9% (328/368) and 37.0% (30/81).

A prior retrospective AI-study with data from two breast centers in the Central Norway Regional Health Authority, where examinations were performed on Siemens machines, reported that 86.8% (653/752) of the screen-detected and 44.9% (92/205) of the interval cancers had an AI score of 10 [25]. This is comparable with the findings in this study of examinations performed on GE machines. There was variability across our two sites, Bergen (85.6%, 327/382) and Stavanger (94.8%, 290/306); however, Stavanger used GE Senographe Pristina from 2018 and Bergen only used GE Senographe Essential. Another study using the AI system from Kheiron Medical Technologies has reported similar results across different mammography vendors [35].

The figures above focus on cancer detection for cases with an AI score of 10 among all cancers. Using all cases with an AI score of 10 in the denominator results in a screen-detected cancer rate of about 6%. This means that 94 out of 100 cases are negative for breast cancer and should be interpreted negative by the radiologists or dismissed at consensus. To do so, knowledge about, e.g., mammographic features of cases with a high AI score but no cancer is required.

A study using data from one of the breast centers included in this study, Rogaland (Stavanger), reported a higher percentage of screen-detected cancers with an AI score of 10 (92.7% versus 89.7%), but a lower percentage for interval cancers with an AI score of 10 (40.0% versus 44.6%) compared to this study [13]. However, their data was from women screened 2010–2018 and included a cancer-enriched sample where 10 negative cases were included for each cancer case. Further, in contrast to the study with a cancer-enriched sample, we found that the highest proportion of an AI score of 10 for examinations prior to interval cancers was observed for VDG3 and not VDG4. Despite including almost 100,000 examinations in this study, the number of interval cancers was lower as the data was from a regular screening setting, without enrichments. The low number of cases weakens the validity and power of our results. All screen-detected cancers among women with VDG4 had an AI score of 10 in the enriched study compared to 94.7% in the present study; however, the cancer-enriched study included only 59 screen-detected cancers for women with VDG4.

AI markings indicate areas suspicious for cancer and are directing the radiologists’ attention. However, the validity of the AI markings is limited explored, and review studies are needed to fill this knowledge gap. A review study from Norway showed that all screen-detected (*n* = 126) and 78% (93/120) of interval cancers with AI score 10 were correctly located by the AI system [36]. Among the interval cancers with AI score 10 and correctly located AI marking, 60% (56/93) were classified as false negative or minimal sign. Informed review studies have classified 20–30% of the screen-detected and interval cancers as missed at prior screening [37, 38]. We found that 44.6% (87/185) of interval cancers had an AI score of 10. A study from Sweden reported that 58% (83/143) of the interval cancers with an AI score of 10 were classified as false negative or minimal sign and could potentially be detected at screening with support from AI in the reading process [16]. The results might indicate a potential for earlier detection of interval cancer when using AI in screen reading.

The scenarios illustrated in Fig. 3 assume AI as a stand-alone interpreter. Interpretations of mammograms without involvement of radiologists require ethical and legal considerations. A study has shown that 23% of the screen-detected cancers were interpreted negatively by one of the two readers in an independent double reading setting [39]. This is important evidence in the discussion we must have before making the decision about how to use AI in the interpretation procedure in screen reading. However, the most common way of using AI is as decision support, which might influence the reading time [40] and recall rate [26, 41].

When comparing the percentage of screen-detected cancers with an AI score of 10 between density categories, the highest percentage was observed for VDG4. Furthermore, the percentage of all examinations, including negative examinations, with an AI score of 10 was also highest for VDG4. This means that specificity was lower for higher AI risk scores for VDG4 cases and the risk of increasing the rate of false-positive screening results must be balanced against possible increased sensitivity for women with extremely dense breasts. Furthermore, we must also bear in mind that about 75% of the screen-detected cancers in our study were among women with VDG2 or VDG3, and only 6% among those with VDG4.

Strengths of our study include the use of data from a regular screening setting, mammographic density measurements from an automated software, and the use of a CE-marked and FDA-approved AI system. Limitations include a relatively low number of cancer cases, especially for VDG1 and VDG4 examinations. The retrospective approach and the limited generalizability to a prospective screening setting also represent shortcomings of our study. Furthermore, we did not have information about the location of the AI marking versus the location of the cancer, which is an important for the validity of the AI system. We did not include interval cancers in the scenarios, which might underestimate the potential of cancer detection by the AI system, as including interval cancers would have added cancer cases. However, this might also have resulted in lower percentages for reduction of screen reading volume in all scenarios.

In conclusion, this study showed promising results for AI to classify cancer cases into different risk score categories regardless of mammographic density. Future prospective studies are needed to support our findings and to establish the evidence needed for the safe implementation of AI in the interpretation process in mammographic screening and as a potential tool to offer personalized screening.

## Supplementary Information

Below is the link to the electronic supplementary material.

Supplementary file1 (PDF 260 KB)

## Abbreviations

AI: Artificial intelligence
DCIS: Ductal carcinoma in situ
GDPR: General data protection regulation
Her2: Human epidermal growth factor receptor 2
IQR: Interquartile range
VDG: Volpara density grade

## Declarations

### Guarantor

The scientific guarantor of this publication is Solveig Hofvind.

### Statistics and biometry

One of the authors (ML) has significant statistical expertise, but no complex statistical methods were necessary for this paper.

### Study subjects or cohorts overlap

A retrospective study using a cancer-enriched sample from Rogaland, 2010–2018, has been published (Koch HW, Larsen M, Bartsch H, Kurz KD, Hofvind S (2023) Artificial intelligence in BreastScreen Norway: a retrospective analysis of a cancer-enriched sample including 1254 breast cancer cases. Eur Radiol 33:3735–3743). This study uses data from a regular screening setting in Rogaland, 2017–2019, so there are some overlapping examinations from 2017 and 2018.

### Methodology

• retrospective

• registry study/observational study

• performed at two breast centers
