# Supplementary material for: AI performance by mammographic density in a retrospective cohort study of 99,489 participants in BreastScreen Norway
Source: Eur Radiol. 2024 Mar 25;34(10):6298–308. doi: 10.1007/s00330-024-10681-z (PMC11399294; doi:10.1007/s00330-024-10681-z)
Supplement: Supplementary file 1 — Supplementary file1 (PDF 260 KB) [file 330_2024_10681_MOESM1_ESM.pdf]

AI performance by mammographic density in a retrospective cohort study of 99,489 participants in BreastScreen Norway

Electronic Supplementary Material (ESM)

Eur Radiol (2024) Burns Bergan M, Larsen M, Moshina N et al.

Supplementary Table 1. Frequencies (n) and percentages (%) with 95% confidence intervals (CI) of all screening examinations, examinations with a negative screening result, screen-detected cancers, interval cancers, and all cancers combined, stratified by AI score 1-10 and breast center (Stavanger and Bergen).

AI Score	All Screening Examinations			Examinations with Negative Screening Result			Screen-detected Cancer			Interval Cancers			Screen-detected and Interval Cancers		
	n	%	(95% CI)	n	%	(95% CI)	n	%	(95% CI)	n	%	(95% CI)	n	%	(95% CI)
Stavanger															
1	9,116	20.9	(20.5-21.3)	9,109	21.1	(20.7-21.5)	0	0.0	(-)	7	7.1	(2.9-14.2)	7	1.7	(0.7-3.5)
2	3,562	8.2	(7.9-8.4)	3,556	8.2	(8.0-8.5)	0	0.0	(-)	6	6.1	(2.3-12.9)	6	1.5	(0.5-3.2)
3	4,732	10.9	(10.6-11.1)	4,729	11.0	(10.7-11.2)	0	0.0	(-)	3	3.1	(0.6-8.7)	3	0.7	(0.2-2.2)
4	4,139	9.5	(9.2-9.8)	4,133	9.6	(9.3-9.8)	2	0.7	(0.1-2.3)	4	4.1	(1.1-10.1)	6	1.5	(0.5-3.2)
5	3,950	9.1	(8.8-9.3)	3,947	9.1	(8.9-9.4)	1	0.3	(<0.1-1.8)	2	2.0	(0.2-7.2)	3	0.7	(0.2-2.2)
6	3,400	7.8	(7.5-8.1)	3,392	7.9	(7.6-8.1)	0	0.0	(-)	8	8.2	(3.6-15.5)	8	2.0	(0.8-3.9)
7	3,307	7.6	(7.3-7.8)	3,301	7.6	(7.4-7.9)	0	0.0	(-)	6	6.1	(0.3-12.9)	6	1.5	(0.5-3.2)
8	3,409	7.8	(7.6-8.0)	3,399	7.9	(7.6-8.1)	4	1.3	(0.4-3.3)	6	6.1	(0.3-12.9)	10	2.5	(1.2-4.5)
9	3,935	9.0	(8.8-9.2)	3,912	9.1	(8.8-9.3)	9	2.9	(1.4-5.5)	14	14.3	(8.0-22.8)	23	5.7	(3.7-8.4)
10	4,053	9.3	(9.0-9.6)	3,721	8.6	(8.4-8.9)	290	94.8	(91.7-97.0)	42	42.9	(32.9-53.3)	332	82.2	(78.1-85.8)
Total	43,603	100.0	(-)	43,199	100.0	(-)	306	100.0	(-)	98	100.0		404	100.0	
Bergen															
1	11,262	20.2	(19.8-20.5)	11,253	20.3	(20.0-20.6)	3	0.8	(0.2-2.3)	6	6.2	(2.3-13.0)	9	1.9	(0.9-3.5)
2	4,075	7.3	(7.1-7.5)	4,072	7.4	(7.1-7.6)	2	0.5	(0.1-1.9)	1	1.0	(<0.1-5.6)	3	0.6	(0.1-1.8)
3	4,830	8.6	(8.4-8.9)	4,824	8.7	(8.5-8.9)	3	0.8	(0.2-2.3)	3	3.1	(0.6-8.8)	6	1.3	(0.5-2.7)
4	4,822	8.6	(8.4-8.9)	4,813	8.7	(8.5-8.9)	2	0.5	(0.1-1.9)	7	7.2	(3.0-14.3)	9	1.9	(0.9-3.5)
5	4,662	8.3	(8.1-8.6)	4,655	8.4	(8.2-8.6)	2	0.5	(0.1-1.9)	5	5.2	(2.0-11.6)	7	1.5	(0.6-3.0)
6	4,261	7.6	(7.4-7.8)	4,254	7.7	(7.5-7.9)	2	0.5	(0.1-1.9)	5	5.2	(2.0-11.6)	7	1.5	(0.6-3.0)
7	4,329	7.8	(7.5-8.0)	4,324	7.8	(7.5-8.0)	4	1.0	(0.3-2.7)	1	1.0	(<0.1-5.6)	5	1.0	(0.3-2.4)
8	5,101	9.1	(8.9-9.4)	5,070	9.2	(8.9-9.4)	17	4.5	(2.6-7.0)	14	14.4	(8.1-23.0)	31	6.5	(4.4-9.0)
9	6,191	11.1	(10.8-11.3)	6,161	11.1	(10.9-11.4)	20	5.2	(3.2-8.0)	10	10.3	(5.1-18.1)	30	6.3	(4.3-8.8)
10	6,353	11.4	(11.1-11.6)	5,981	10.8	(10.5-11.1)	327	85.6	(81.7-89.0)	45	46.4	(36.2-56.8)	372	77.7	(73.7-81.3)
Total	55,886	100.0	(-)	55,407	100.0	(-)	382	100.0	(-)	97	100.0	(-)	479	100.0	(-)

Supplementary Table 2. Histopathological characteristics of screen-detected and interval DCIS with AI score 10 and AI score 1-9.

	Screen-detected DCIS				Interval DCIS			
	AI score 10, n = 115		AI score 1-9, n = 4		AI score 10, n = 6		AI score 1-9, n = 8	
	n	%	n	%	n	%	n	%
Tumor diameter (mm), median (IQR)	20 (12-35)		12 (10-15)		15.5 (9-30)		40 (31.5-66)	
Information not available	12		1		-		-	
Van Nuys Grade								
Grade 1	19	16.5	2	50.0	2	33.3	2	25.0
Grade 2	16	13.9	0	0.0	1	16.7	0	0.0
Grade 3	80	69.6	2	50.0	3	50.0	6	75.0

Supplementary Table 3. Frequencies (n) and percentages (%) with 95% confidence intervals (CI) of screening examinations with AI score 1-10, stratified by volumetric breast density (VDG1, VDG2, VDG3, and VDG4) and breast center (Stavanger and Bergen).

AI Score	VDG1			VDG2			VDG3			VDG4		
	n	%	(95% CI)	n	%	(95% CI)	n	%	(95% CI)	n	%	(95% CI)
Stavanger												
1	2,514	29.4	(28.4-30.4)	4,052	20.6	(20.1-21.2)	1,965	16.2	(15.6-16.9)	585	17.9	(16.6-19.2)
2	538	6.3	(5.8-6.8)	1,511	7.7	(7.3-8.1)	1,171	9.6	(9.1-10.2)	342	10.4	(9.4-11.5)
3	1,032	12.1	(11.4-12.8)	2,209	11.3	(10.1-11.7)	1,221	10.1	(9.5-10.6)	270	8.2	(7.3-9.2)
4	803	9.4	(8.8-10.0)	1,895	9.7	(9.2-10.1)	1,181	9.7	(9.2-10.3)	260	7.9	(7.0-8.9)
5	732	8.6	(8.0-9.2)	1,776	9.0	(8.6-9.5)	1,189	9.8	(9.3-10.3)	253	7.7	(6.8-8.7)
6	614	7.2	(6.6-7.7)	1,510	7.7	(7.3-8.1)	1,044	8.6	(8.1-9.1)	232	7.1	(6.2-8.0)
7	565	6.6	(6.1-7.2)	1,517	7.7	(7.4-8.1)	966	8.0	(7.5-8.5)	259	7.9	(7.0-8.9)
8	563	6.6	(6.1-7.1	1,566	8.0	(7.6-8.4)	1,000	8.2	(7.8-8.7)	280	8.5	(7.6-9.6)
9	620	7.2	(6.7-7.8)	1,740	8.9	(8.5-9.3)	1,197	9.9	(9.3-10.4)	378	11.5	(10.5-12.7)
10	575	6.7	(6.2-7.3)	1,856	9.5	(9.0-9.9)	1,205	9.9	(9.4-10.5)	417	12.7	(11.6-13.9)
Total	8,556	100.0	(-)	19,632	100.0	(-)	12,139	100.0	(-)	3,276	100.0	(-)
Bergen												
1	4,008	34.5	(33.6-35.4)	5,183	17.4	(17.0-17.9)	1,567	13.3	(12.7-13.9)	504	18.2	(16.8-19.7)
2	732	6.3	(5.9-6.8)	2,128	7.2	(6.9-7.5)	971	8.2	(7.8-8.8)	244	8.8	(7.8-9.9)
3	1,090	9.4	(8.9-9.9)	2,575	8.7	(8.3-9.0)	925	7.9	(7.3-8.4)	240	8.7	(7.6-9.8)
4	946	8.1	(7.6-8.7)	2,675	9.0	(8.7-9.3)	1,007	8.5	(8.9-9.1)	194	7.0	(6.1-8.0)
5	831	7.2	(6.7-7.6)	2,524	8.5	(8.2-8.8)	1,098	9.3	(8.8-9.8)	209	7.5	(6.6-8.6)
6	693	6.0	(5.5-6.4)	2,394	8.1	(7.6-8.4)	986	8.4	(7.9-8.9)	188	6.8	(5.9-7.8)
7	744	6.4	(6.0-6.9)	2,379	8.0	(7.7-8.3)	1,019	8.7	(8.1-9.2)	187	6.7	(5.8-7.7)
8	742	6.4	(5.9-6.8)	2,908	9.8	(9.5-10.1)	1,194	10.1	(9.6-10.7)	257	9.3	(8.2-10.4)
9	927	8.0	(7.5-8.5)	3,398	11.4	(11.1-11.8)	1,513	12.8	(12.2-13.5)	353	12.7	(11.5-14.0)
10	909	7.8	(7.3-8.3)	3,549	11.9	(11.6-12.3)	1,500	12.7	(12.1-13.3)	395	14.3	(13.0-15.6)
Total	11,622	100.0	(-)	29,713	100.0	(-)	11,780	100.0	(-)	2,771	100.0	(-)

Supplementary Table 4. Frequencies (n) and percentages (%) of interval cancers with AI score 1-10, stratified by volumetric breast density (VDG1, VDG2, VDG3 and VDG4)*.

AI Score	VDG1		VDG2		VDG3		VDG4	
	n	%	n	%	n	%	n	%
1	1	11.1	6	7.4	6	7.5	-	-
2	-	-	2	2.5	5	6.3	-	-
3	-	-	3	3.7	2	2.5	1	4.0
4	-	-	5	6.2	4	5.0	2	8.0
5	-	-	6	7.4	1	1.3	-	-
6	2	22.2	6	7.4	3	3.8	2	8.0
7	-	-	3	3.7	3	3.8	1	4.0
8	1	11.1	9	11.1	7	8.8	3	12.0
9	2	22.2	11	13.6	7	8.8	4	16.0
10	3	33.3	30	37.0	42	52.5	12	48.0
Total	9	100.0	81	100.0	80	100.0	25	100.0

*Due to the small numbers, 95% confidence intervals or p-values were not estimated
